# Current Status and Perspectives of Human Mesenchymal Stem Cell Therapy

**DOI:** 10.1155/2019/4762634

**Published:** 2019-03-07

**Authors:** Jane Ru Choi, Kar Wey Yong, Hui Yin Nam

**Affiliations:** ^1^University of British Columbia, Department of Mechanical Engineering, 2054-6250 Applied Science Lane, Vancouver, BC, Canada V6T 1Z4; ^2^University of British Columbia, Centre for Blood Research, Life Sciences Centre, 2350 Health Sciences Mall, Vancouver, BC, Canada V6T 1Z3; ^3^University of Calgary, Pharmaceutical Production Research Facility, Department of Chemical and Petroleum Engineering, Schulich School of Engineering, 2500 University Drive NW, Calgary, AB, Canada T2N 1N4; ^4^University of Calgary, BioMEMS and Bioinspired Microfluidic Laboratory, Department of Mechanical and Manufacturing Engineering, Schulich School of Engineering, 2500 University Drive NW, Calgary, AB, Canada T2N 1N4; ^5^Tissue Engineering Group (TEG), National Orthopaedic Centre of Excellence in Research and Learning (NOCERAL), Department of Orthopaedic Surgery, Faculty of Medicine, University of Malaya, Lembah Pantai, 50603 Kuala Lumpur, Malaysia

Human mesenchymal stem cells (MSCs) hold tremendous potential in cell-based therapies and regenerative medicine [1]. These cells can be readily isolated from multiple sources including bone marrow, fat, umbilical cord, and menstrual blood, enabling the ease of their procurement [2, 3]. They are relatively free from ethical concerns and have the capability of secreting bioactive factors and differentiating into specialized cells of the tissues they reside in [4, 5]. These make them a prospective medical therapy to treat diseases such as musculoskeletal, ischemic, and respiratory diseases [6–8]. While numerous preclinical and clinical studies suggested the therapeutic potential of MSCs in various clinical fields, multiple challenges are yet to be addressed to achieve successful clinical translations [9, 10]. This special issue highlights the recent advances in the clinical use of MSCs, which allows better understanding of their therapeutic impact. The development of new strategies to improve their therapeutic effects especially in the aspects of bioprocessing, safety and efficacy assessment, cell administration route, and delivery strategies has been discussed.

A total of 14 articles introduced the use of MSCs for the treatment of multiple types of diseases, including musculoskeletal, ischemic, respiratory, neurological, autoimmune, and retinal degeneration diseases. A number of comprehensive review articles highlighted the current status and perspectives of MSC therapy in diverse scientific areas including tissue engineering, dentistry, and ischemic and autoimmune diseases. For example, R. E. B. Fitzsimmons et al. reviewed the recent advances of MSC therapy in tissue engineering. The history of MSCs, their sources, and the challenges remained in cell bioprocessing, including cell isolation, cell expansion, and downstream processes for clinical applications, were comprehensively discussed. A. G. Paz et al. reviewed the use of stem cell therapy in dentistry. Various sources and unique properties of MSCs and their potential clinical applications in the field of dentistry, including regeneration of tooth, salivary glands, and mandibular condyle, were briefly discussed.

W. Chen et al. reviewed the use of MSCs for the treatment of primary Sjögren's syndrome (pSS). MSCs are known to suppress autoimmunity and improve the secretory function of the salivary gland in patients with pSS by upregulating regulatory T cells, inactivating proinflammatory T cells, and differentiating themselves into salivary epithelial cells. Furthermore, K. W. Yong et al. discussed the potential therapeutic roles of human MSCs in ischemic diseases. Generally, MSCs repair ischemic tissues and restore the tissue function mainly via angiogenesis and immunomodulation through paracrine secretion of bioactive factors. However, there are some remaining formidable challenges, including poor engraftment and persistence in the host and limited accessibility of the functional *in vitro* ischemic disease model. The possible solutions and future perspectives were discussed for ischemic disease therapy. Moreover, B. Xie et al. performed meta-analysis on randomized controlled clinical trials of critical limb ischemia (CLI) to assess the efficacy and safety of human autologous stem cell therapy, including bone marrow-derived MSCs (BMMSCs) in CLI. It was found that stem cell therapy reduces the ulcer size and the limb amputation rate, improves angiogenesis, and restores limb function and limb perfusion without any adverse effects. The authors also revealed that cell administration route, cell dosage, and cell type are critical factors in stem cell therapy of CLI.

A number of research articles in this issue have reported the therapeutic effects of MSCs in several diseases, such as ischemic stroke, neurological disease, acute lung injury, retinal degeneration, and cartilage repair. Y. Guan et al. demonstrated the use of BMMSCs to reduce the overall number of infiltrated monocytes (Ly6C^+^ cells) in rat ischemic stroke models induced by distal middle cerebral artery occlusion. Interestingly, there was an increase in the proportions of Ly6C^+^ cells that express brain-derived neurotrophic factor or proinflammatory cytokines (tumor necrosis factor-*α* or interleukin-1*β*) in the ischemic areas, which could contribute to the neuroprotection and recovery of ischemic stroke. In addition, D. Y. Lee et al. reported the use of BMMSCs to suppress inflammatory activity of activated microglia for the treatment of neurological diseases. Interestingly, coculturing BMMSCs with lipopolysaccharide-stimulated primary rat microglia increases the migration of BMMSCs to the microglia and hence reduces the inflammatory response. The outcome of this study will improve understanding of the relationship between activated microglia and MSCs, which may lead to a new therapeutic strategy using MSCs for neurological diseases.

In another study, H. Ren et al. compared the therapeutic effects of umbilical cord- and menstrual blood-derived MSCs using the mouse acute lung injury models. The authors found that both sources of MSCs are able to repair the lung tissues by suppressing the inflammation via paracrine release of bioactive factors. Interestingly, umbilical cord-derived MSCs secreted higher levels of anti-inflammatory cytokines (keratinocyte growth factor and interleukin-10) as compared to those derived from menstrual blood, resulting in a better recovery of the lung tissues. A. Barzelay et al. demonstrated that adipose-derived MSCs are able to migrate to retinal pigment epithelial cells to protect them against cell death induced by oxidative stress. This finding suggests that MSCs are extremely useful in future retinal degeneration treatment. Besides that, S. Zheng et al. reported that type 3 transforming growth factor-beta receptor (TGF-*β*R3) plays a vital role in regulating chondrogenic differentiation of BMMSCs. Silencing the activity of TGF-*β*R3 using TGF-*β*R3 RNA interference increased TGF-*β*-smad2/3 signaling and hence enhanced the chondrogenic differentiation of MSCs. It was suggested that MSCs could be modified by TGF-*β*R3 knockdown, which serves as a potential strategy for cartilage regeneration.

Other research articles have suggested some effective methods to improve therapeutic efficacy of MSCs. For instance, S. Baig et al. used chitosan nanoparticles as a carrier for *Thymus serpyllum* extract to evaluate its protective effects on BMMSCs against oxidative stress. This herb extract was able to reduce oxidative stress-induced apoptosis of MSCs as it contains antioxidant compounds (*e.g.*, polyphenol). This finding suggests that chitosan nanoparticles can be used to control the release of *Thymus serpyllum* extract for improving the survival rate of MSC transplant and hence enhancing therapeutic efficacy of MSCs. Furthermore, X. Yang et al. investigated the effects of hydroxyapatite nanoparticles (HA NPs) on osteogenesis of BMMSCs. It was found that HA NPs enhanced osteogenic potential of MSCs in a size-dependent manner. HA NPs with sizes of 50 nm and 100 nm were particularly effective in promoting osteogenic differentiation of MSCs, which appear promising for bone regeneration. S. Sancilio et al. developed a scaffold made up of alginate and HA NPs for encapsulating dental pulp-derived MSCs to create a dental pulp construct. This scaffold supported osteogenic differentiation of MSCs and enhanced bone mineralization, suggesting its potential use in developing dental pulp tissue construct for tooth regeneration. Additionally, H. Y. Nam et al. applied uniaxial cyclic stretching on BMMSCs to induce tenogenic differentiation. It was observed that 8% tensile strain at 1 Hz specifically mediates tenogenic differentiation of MSCs for tendon regeneration.

In summary, this special issue has provided unprecedented insights into the roles of human MSC in treating numerous diseases and highlighted the remaining challenges and possible strategies to enhance their therapeutic potential. Despite significant advancements in MSC therapy, a deeper understanding of the nature, function, mechanism, mode of isolation, and route of administration as well as experimental handling of MSCs is critical to improve the therapeutic efficacy of MSCs. While there are many obstacles remain to be overcome, we envision that the therapeutic potential of MSCs will attract further research investments in this area to resolve the challenges and improve the effectiveness of medical treatment.
